# The differential ability of two species of seagrass to use carbon dioxide and bicarbonate and their modelled response to rising concentrations of inorganic carbon

**DOI:** 10.3389/fpls.2022.936716

**Published:** 2022-09-29

**Authors:** Stephen Christopher Maberly, Andrew W. Stott, Brigitte Gontero

**Affiliations:** ^1^ UK Centre for Ecology & Hydrology, Lancaster Environment Centre, Lancaster, United Kingdom; ^2^ Aix Marseille Univ, CNRS, BIP, UMR 7281, IMM, Marseille, France

**Keywords:** climate change, CO_2_ concentrating mechanisms (CCMs), ocean acidification, *Posidonia oceanica*, rising CO_2_, seagrass, *Zostera marina*

## Abstract

Seagrass meadows are one of the most productive ecosystems on the planet, but their photosynthesis rate may be limited by carbon dioxide but mitigated by exploiting the high concentration of bicarbonate in the ocean using different active processes. Seagrasses are declining worldwide at an accelerating rate because of numerous anthropogenic pressures. However, rising ocean concentrations of dissolved inorganic carbon, caused by increases in atmospheric carbon dioxide, may benefit seagrass photosynthesis. Here we compare the ability of two seagrass from the Mediterranean Sea, *Posidonia oceanica* (L.) Delile and *Zostera marina* L., to use carbon dioxide and bicarbonate at light saturation, and model how increasing concentrations of inorganic carbon affect their photosynthesis rate. pH-drift measurements confirmed that both species were able to use bicarbonate in addition to carbon dioxide, but that *Z. marina* was more effective than *P. oceanica*. Kinetic experiments showed that, compared to *Z. marina*, *P. oceanica* had a seven-fold higher affinity for carbon dioxide and a 1.6-fold higher affinity for bicarbonate. However, the maximal rate of bicarbonate uptake in *Z. marina* was 2.1-fold higher than in *P. oceanica*. In equilibrium with 410 ppm carbon dioxide in the atmosphere, the modelled rates of photosynthesis by *Z. marina* were slightly higher than *P. oceanica*, less carbon limited and depended on bicarbonate to a greater extent. This greater reliance by *Z. marina* is consistent with its less depleted ^13^C content compared to *P. oceanica*. Modelled photosynthesis suggests that both species would depend on bicarbonate alone at an atmospheric carbon dioxide partial pressure of 280 ppm. *P. oceanica* was projected to benefit more than *Z. marina* with increasing atmospheric carbon dioxide partial pressures, and at the highest carbon dioxide scenario of 1135 ppm, would have higher rates of photosynthesis and be more saturated by inorganic carbon than *Z. marina*. In both species, the proportional reliance on bicarbonate declined markedly as carbon dioxide concentrations increased and in *P. oceanica* carbon dioxide would become the major source of inorganic carbon.

## Introduction

Seagrasses are marine angiosperms from four families (or five if the brackish water Ruppiaceae are included), all from the monocot order Alismatales, that evolved ultimately from land plants, probably *via* freshwater ancestors, and first returned to the sea in the Cretaceous period, around 140 to 100 million years ago (Les et al., 1997; Wissler et al., 2011; Les and Tippery, 2013). Seagrass meadows deliver many globally-important and locally-important ecosystem goods and services (Larkum et al., 2006). Globally, they cover an area of between 0.15 to 4.32 10^6^ km^2^ (Duarte, 2017) and are one of the most productive ecosystems on the planet with an average primary productivity of 394 to 1200 g C m^-2^ y^-1^ (Van Der Heijden and Kamenos, 2015; Raven, 2018), a global productivity of 0.06 to 1.94 Pg C y^-1^ (Raven, 2018) and are responsible for burying about 20% of all oceanic carbon (Duarte et al., 2005). Locally, they support food-webs and commercial fisheries (Unsworth et al., 2019), stabilize sediments, cycle nutrients and provide habitats for many species (Larkum et al., 2006; Unsworth et al., 2019). They also improve water quality, including the removal of bacterial pathogens (Lamb et al., 2017).

The transition of plants from land back to water, traded-off problems of water-availability for problems of carbon and light availability (Maberly, 2014; Maberly and Gontero, 2017). Even at current equilibrium with ~410 ppm CO_2_ in the atmosphere, concentrations of dissolved CO_2_ in sea water (10 to 18 µmol L^-1^ at 30 to 10°C) are only slightly above the presumed Michaelis-Menten constant (K_M_ for CO_2_) of ribulose 1,5-bisphosphate carboxylase/oxygenase (Rubisco) which is in the range of 8 to 14 µmol L^-1^ for terrestrial angiosperms (Galmes et al., 2014; Galmes et al., 2016) and substantially below the mean of four seagrass species at 29 µmol L^-1^ (Capó-Bauçà et al., 2022). Passive uptake of CO_2_ is therefore likely to be highly carbon-limited under these conditions, especially since the rate of CO_2_ diffusion into a leaf, through substantial external boundary layers, is about 10 000-times lower in water than in air. Furthermore, inorganic carbon can become depleted in highly productive seagrass beds during the day when biological demand exceeds environmental supply, reducing the concentration of CO_2_ to below air equilibrium, elevating pH and inevitably decreasing the concentration of $\text{HC}{\text{O}}_{3}^{-}$ (Invers et al., 2001; Hendriks et al., 2014; Koopmans et al., 2020). Furthermore, oxygen maxima of 377 µmol L^-1^ (~130% air saturation) and CO_2_ minima of 193 ppm on a volume basis (~48% air saturation) have been recorded during the day within a *Z. marina* meadow off the coast of Virginia, USA (Berg et al., 2019). An even higher oxygen saturation value of about 180% has been recorded in similar meadows (Long et al., 2020) and gas phase oxygen concentrations in internal lacunae may be even higher. These diurnal changes have the potential to stimulate photorespiration and limit photosynthesis to an even greater extent than under atmospheric equilibrium conditions. Interestingly, Rubisco from seagrasses had a lower catalytic efficiency (maximum catalytic rate divided by K_M_) for oxygen than terrestrial plants or freshwater plants (Capó-Bauçà et al., 2022) which might be an adaptation to reduce photorespiration and perhaps represents a trade-off with their higher K_M_ for CO_2_.

In response to inorganic carbon limitation, many aquatic plants have evolved a range of carbon acquisition strategies (Klavsen et al., 2011) some of which are based on active CO_2_ concentrating mechanisms (CCMs) (Larkum et al., 2017; Maberly and Gontero, 2017; Maberly and Gontero, 2022)). Many, but not all, species of seagrasses, have CCMs that exploit the high concentration of bicarbonate ( $\text{HC}{\text{O}}_{3}^{-}$ ; ~2 mmol L^-1^) in the ocean (Beer et al., 2002; Larkum et al., 2006;Koch et al., 2013; Borum et al., 2016; Larkum et al., 2017). Since seagrass cells have a membrane potential of about -160 to -170 mV [i.e. negative inside the cell; (Rubio and Fernández, 2019)] and the plasmalemma is relatively impermeable to ions, $\text{HC}{\text{O}}_{3}^{-}$ cannot enter the leaf passively and $\text{HC}{\text{O}}_{3}^{-}$ use has to be an active process (Raven, 1970). In some freshwater plants, in addition to $\text{HC}{\text{O}}_{3}^{-}$ use, biochemical CCMs involving C_4_ photosynthesis and Crassulacean Acid Metabolism are found (Maberly and Gontero, 2018), however, there is little evidence that they are important in seagrasses (Koch et al., 2013; Larkum et al., 2017). Carbonic anhydrase catalyzes the interconversion of CO_2_ and $\text{HC}{\text{O}}_{3}^{-}$ and is present ubiquitously (Supuran, 2018; Jensen et al., 2020) and is involved in CCMs. An external, periplasmic, carbonic anhydrase, between the plasmalemma and cell wall, is frequently involved in facilitating inorganic carbon uptake (James and Larkum, 1996; Larsson and Axelsson, 1999; Moroney et al., 2011; Tachibana et al., 2011; Van Hille et al., 2014; Fernandez et al., 2018). Solute carrier proteins from family 4 (SLC4) are involved in anion exchange (Romero et al., 2013) and have been implicated in facilitating $\text{HC}{\text{O}}_{3}^{-}$ uptake in a range of aquatic species (Drechsler et al., 1993; Bjork et al., 1997; Nakajima et al., 2013; Fernandez et al., 2014; Poliner et al., 2015; Huang et al., 2020).

Seagrasses are threatened worldwide by direct and indirect human interference that includes physical disturbance, pollution and nutrient enrichment, increased sediment load and climate change (Orth et al., 2006; Waycott et al., 2009). Globally, seagrass meadows have been estimated to be declining by 110 km^2^ y^-1^ since 1980 and the rate of loss is accelerating (Waycott et al., 2009). Increasing CO_2_ in the atmosphere is causing oceanic surface water temperature to increase (IPCC, 2021). It also causes the concentrations of CO_2_ and $\text{HC}{\text{O}}_{3}^{-}$ to increase, while pH is declining (Raven et al., 2005; IPCC, 2021). Ocean acidification can be harmful, especially to those species that produce calcite exoskeletons as it causes the concentration of carbonate to decline (Doney et al., 2009). However, the increasing concentration of CO_2_ has the potential to favor seagrass photosynthesis by reducing carbon limitation (Hall-Spencer et al., 2008; Burnell et al., 2014; Garrard and Beaumont, 2014; Pajusalu et al., 2016). Borum et al. (Borum et al., 2016) found differences among nine Australian seagrasses in the extent to which their photosynthesis was stimulated by increasing CO_2_.

We studied two species from the Mediterranean Sea. *Posidonia oceanica* (L.) Delile belongs to the family Posidoniaceae and is endemic to the Mediterranean Sea where it is an important ‘ecological engineer’ (Personnic et al., 2014). *Zostera marina* L. belongs to the family Zosteraceae and is one of the most intensively studied and widespread species, found in the North Atlantic, North Pacific and Arctic Oceans and also the Mediterranean Sea (Olsen et al., 2016). Both species have been shown to use $\text{HC}{\text{O}}_{3}^{-}$ as well as CO_2_ (see references below). The aim of this work was firstly to determine the uptake characteristics of CO_2_ and $\text{HC}{\text{O}}_{3}^{-}$ for these two species of seagrass using pH-drift, stable isotope discrimination, measurement of photosynthesis kinetics and analysis of protein sequences. By characterizing the uptake kinetics of $\text{HC}{\text{O}}_{3}^{-}$ from CO_2_, separately, the second aim was to evaluate the response of the two species to rising CO_2_ using a model based on the kinetic measurements and scenarios of future increases in atmospheric CO_2_.

## Materials and methods

### Sampling sites

Two species of seagrass were collected in July 2019 from the Mediterranean Sea near Marseille in the South of France. *P. oceanica* was collected from St Cyr sur Mer (43° 9.54’N, 5° 37.283’E) at a depth of 18 m. *Z. marina* was collected from Etang de Thau (43°, 25.1607’N, 3° 36.0107’E) at a depth of 1 m. The salinity was about 38 at both sites and the water temperature was between 21 and 25°C. Plants were transported back to the laboratory in a cool box and kept in an incubator (Innova 4230, New Brunswick Scientific, Edison, New Jersey, USA) at 20°C in natural seawater under continuous illumination from Grolux fluorescent tubes producing about 70 µmol m^-2^ s^-1^ photosynthetically active radiation (PAR, 400 to 700 nm; Q201, Macam Photometric, Livingstone, UK) and used within two days of collection. Material for the pH-drift and stable carbon isotope analysis was collected two years earlier in July 2017 and used immediately. In 2017, *Z. marina* was collected from Etang de Thau as in 2019, but in 2017, *P. oceanica* was collected from the Bay of Marseille (43° 16.38’N, 5° 20.43’E) at a depth of 12 m.

### Carbon uptake kinetics

Rates of net photosynthesis were measured as oxygen evolution at 20°C in an electrode chamber (Oxygraph, Hansatech Instruments, Norfolk, UK). The chamber was illuminated with a 35 W halogen GU10C lamp behind a hot-mirror cut-off filter at 750-1100 nm (HMC-1033, UQG Cambridge, UK) to minimize heat input to the chamber. The leaves received ~400 µmol m^-2^ s^-1^ PAR, which preliminary experiments had shown to be saturating, but not photo-inhibiting. Leaves of 25 mm length and 10 mm width (*P. oceanica*) or 28 mm length and 5 mm width (*Z. marina*), were rinsed for a few seconds in artificial seawater (Kester et al., 1967) without NaHCO_3_, boric acid, sodium fluoride or strontium chloride (**Supplementary Table 1**) and were then incubated for fifteen minutes at 20°C in artificial seawater with 0.2 mM $\text{HC}{\text{O}}_{3}^{-}$ , added as NaHCO_3_, and bubbled with air. The leaf was placed in the oxygen electrode with 2 mL of the same solution and an initial oxygen concentration of about 70% saturation produced by briefly bubbling with nitrogen, and the rate of oxygen evolution was measured. A predetermined volume of 0.1 or 1 mol L^-1^ HCl was then injected into the chamber to lower pH (on the NBS scale) to pH 7 generating increased concentrations of CO_2_ at very similar concentrations of $\text{HC}{\text{O}}_{3}^{-}$ (**Supplementary Table 2**) and the rate was re-measured. The leaf was then removed from the electrode and incubated for 15 minutes at the next $\text{HC}{\text{O}}_{3}^{-}$ concentration and rates measured following the same procedure. Five NaHCO_3_ concentrations were used, up to a maximum of 5 mmol L^-1^. Each measurement continued until a constant slope was achieved, which typically lasted for around three to six minutes. No additional buffers were used because our preliminary experiments showed that the inclusion of 10 mM HEPES increased the K½ for $\text{HC}{\text{O}}_{3}^{-}$ 20-fold in *P. oceanica* and 6-fold in *Z. marina*. This procedure produced different concentrations of $\text{HC}{\text{O}}_{3}^{-}$ at air-equilibrium CO_2_ and higher concentrations of CO_2_ at near-constant $\text{HC}{\text{O}}_{3}^{-}$ concentration following addition of acid. Concentrations of CO_2_ varied between 0.013 and 0.31 mM and $\text{HC}{\text{O}}_{3}^{-}$ concentrations varied between 0.20 and 3.93 mM (**Supplementary Table 2**). The measurements were made in triplicate.

At the end of the sequence, the leaves were blotted quickly and weighed to determine the fresh weight. The leaves were photographed and leaf area estimated using AreaAna software (Huazhong University of Sciences and Technology, China). A subsample was ground in a mortar and pestle and the pigments extracted in 100% ethanol in the dark overnight at 4°C. Following centrifugation and measurement of optical density in a spectrophotometer, chlorophyll *a* and *b* were estimated using the equations in (Porra et al., 1989). Seventeen different leaves of each species were used to determine the fresh weight to dry weight ratio. Fresh weight was measured as above and dry weight after drying for 24 hours at 80°C.

The response of the rate of net photosynthesis to the concentration of CO_2_ and $\text{HC}{\text{O}}_{3}^{-}$ was fitted to a modified Michaelis-Menten equation. The model (Clement et al., 2016), assumes separate uptake of these two forms of inorganic carbon with different K_½_ and compensation concentrations but a common total maximum uptake rate for each carbon species:


(Eqn 1) $$Net\;rate\;of\;photosynthesis=\left(\frac{\alpha *Total\;V_{net}^{max}*(CO_{2}-CP^{C})}{K_{1/2}^{C}+(CO_{2}-CP^{C})}\right)+\left(\frac{\left(1-\alpha )*Total\;V_{net}^{max}*(HCO_{3}^{-}-CP^{B}\right)}{K_{1/2}^{B}+(HCO_{3}^{-}-CP^{B})}\right)$$

Where rates are expressed in µmol O_2_ g^-1^ FW h^-1^ and concentrations in µmol L^-1^:

$Total\;V_{net}^{max}$ = the total maximum rate of net photosynthesis for CO_2_ plus $\text{HC}{\text{O}}_{3}^{-}$

α = the proportion of $V_{net}^{max}$ resulting from CO_2_ uptake (unitless)

CO_2_ = the concentration of CO_2_

*CP^C^* = the CO_2_ compensation concentration

$K_{1/2}^{C}$ = the concentration of CO_2_ yielding half-maximal rates of net photosynthesis

$\text{HC}{\text{O}}_{3}^{-}$ = the concentration of $\text{HC}{\text{O}}_{3}^{-}$

*CP^B^* = the $\text{HC}{\text{O}}_{3}^{-}$ compensation concentration

$K_{1/2}^{B}$ = the concentration of $\text{HC}{\text{O}}_{3}^{-}$ yielding half-maximal rates of net photosynthesis

The model is similar to that developed for *Z. marina* by McPherson et al. (2015) but differs as we introduced compensation concentrations for both inorganic carbon sources and linked maximum rates of CO_2_ and $\text{HC}{\text{O}}_{3}^{-}$ uptake with a common maximum rate, $V_{net}^{max}$ . The true K_½_ concentration was calculated from the modelled K_½_ plus the compensation concentration. The slope was calculated from the ratio of $V_{net}^{max}$ to the modelled K_½_ concentration. The best fit of the model parameters to the data for each replicate was obtained by minimizing the residual sum of squares of the difference between the measured and modelled rate of net photosynthesis using ‘Solver’ in Excel for Microsoft 365. Different starting conditions were used to guard against finding a local best-fit. Mean and standard deviation of the model parameters were calculated from the replicates. An example of the results and fits for one replicate of *Z. marina* is shown in **Supplementary Figure 1**.

### Estimated rates of photosynthesis under a range of atmospheric CO_2_ partial pressures

The kinetic data were used to forecast rates of photosynthesis, at 20°C and light saturation, for variable concentrations of CO_2_ at a constant seawater carbonate alkalinity of 2.29 mequiv L^-1^ and a salinity of 36. CO_2_-dependent rates were estimated for CO_2_ concentrations from 0.0004 to 0.06 mmol L^-1^ and $\text{HC}{\text{O}}_{3}^{-}$ dependent rates were estimated for the range of $\text{HC}{\text{O}}_{3}^{-}$ concentrations, 0.63 to 2.26 mmol L^-1^, that corresponded to the CO_2_ concentrations. Rates were extracted at CO_2_ partial pressures in the atmosphere of 280 ppm (pre-industrial), 410 ppm (contemporary) and four projections to 2100 based on the shared socioeconomic pathway (SSP) scenarios used in the Sixth Assessment Report by the IPCC (2021; (IPCC, 2021)): 446 ppm for SSP1-2.6, 603 ppm for SSP2-4.5, 867 ppm for SSP3-7.0 and 1135 ppm for SSP5-8.5 (Meinshausen et al., 2020). The calculated pH values and concentrations of CO_2_ and $\text{HC}{\text{O}}_{3}^{-}$ at 20°C for the scenarios are given in **Supplementary Table 3**. The absolute concentration of $\text{HC}{\text{O}}_{3}^{-}$ increases more than CO_2_ at equilibrium with increasing atmospheric CO_2_ partial pressure but the proportion of $\text{HC}{\text{O}}_{3}^{-}$ to CO_2_ decreases. The percent contribution of CO_2_-dependent and $\text{HC}{\text{O}}_{3}^{-}$ dependent rates to the sum of both rates (Total $V_{net}^{max}$ ) was calculated.

### pH-drift

The maximum pH achieved in pH-drift experiments was determined by placing about 0.17 g fresh weight of leaf in 15 mL plastic Falcon tubes with a total volume of 16.3 mL containing 14 mL of natural seawater that had been passed through a 0.45 µm filter. There was consequently a gas headspace of about 2.3 mL. The tubes were capped and placed horizontally in an incubator (Innova 4230, New Brunswick Scientific, Edison, New Jersey, USA) at 20°C under continuous illumination from Grolux fluorescent tubes producing about 70 µmol m^-2^ s^-1^ PAR and shaken at 60 rpm. A combination pH-electrode (Hanna HI 1043, Woonsocket, Rhodes Island (USA) was calibrated with buffers at pH 7 and 10 on the NBS scale and pH was measured after 24 hours and roughly after every 12 hours until a maximum pH had been reached. Four replicates per species were used. A control tube without any leaves was also incubated. Alkalinity was measured at the end of the experiment by Gran titration (Mackereth et al., 1978).

### Stable carbon isotope analysis

To determine isotope values of the dissolved inorganic carbon (DIC) source, ten mL of seawater were collected *in situ* at the two collection sites in evacuated exetainers containing phosphoric acid and stored inverted before analysis (Waldron et al., 2007). In the laboratory, four mL of helium (99.999%) were injected into the headspace of each exetainer to over-pressurize. After shaking and 30 minutes equilibration, a 40 µL gas sample was removed for analysis in an Isoprime Ltd Tracegas Preconcentrator (Isoprime, Manchester, UK) coupled to an Isoprime Ltd isotope ratio mass spectrometer. Pulses of known reference CO_2_ and blanks were run prior to each batch.

Material freshly collected from the sea was dried at 80°C and stored in aluminum foil prior to analysis. Small amounts of the plant were re-dried at 105°C, aliquots sealed into 6 x 5 mm tin capsules and loaded into an autosampler (Eurovector Elemental Analyser, Eurovector, Milano Italy) coupled in-line to a stable isotope ratio mass spectrometer. Each sample was combusted at 1020°C with a pulse of oxygen and products were carried by a flow of helium through a reduction reactor containing copper wire at 650°C and dried with magnesium perchlorate. N_2_ and CO_2_ were separated by a packed GC column and delivered, *via* an ‘open-split’ to the isotope ratio mass spectrometer. Values were compared to pulses of CO_2_ reference gas and to a solid working standard of known isotopic composition. Stable carbon data were expressed in the delta notation (δ^13^C) relative to the Vienna Pee Dee Belemnite (VPDB) standard (Eqn 2). Stable isotope methods for inorganic and organic C were accredited to UKAS ISO17025. The δ^13^C values of potential inorganic carbon sources, CO_2_ and $\text{HC}{\text{O}}_{3}^{-}$ , were calculated from the measured δ^13^C values of DIC using the temperature-dependent equations of (Mook et al., 1974). Discrimination (Δ) of the leaf δ^13^C values was calculated following (Maberly et al., 1992) (Eqn 3) against CO_2_ and $\text{HC}{\text{O}}_{3}^{-}$ and against the proportional amounts of CO_2_ and $HCO_{3}^{-}$ used in ambient conditions that were estimated from the photosynthesis kinetics.


(Eqn 2) $$\delta^{13}C=(R_{sample}-R_{VPDB}/R_{VPDB})\times 1000$$

Where R_Sample_ and R_VPDB_ are the ^13^C:^12^C ratios of the sample and standard respectively.


(Eqn 3) $$\Delta =(\delta_{Source}-\delta_{Plant})/(1+\delta_{Plant})$$

### Inorganic carbon speciation

Inorganic carbon concentrations were calculated using the CO2SYS_calc program in Excel (Pierrot et al., 2006) and converted from molal to molar using a seawater density at 20°C of 1.025 kg L^-1^ for artificial seawater at a salinity of 36 used in the kinetic experiments and 1.027 for the natural seawater with a salinity of 38 used in the pH-drift experiments and analysis of the stable isotope results.

### Sequence analysis

Since the genome of *Z. marina* has been sequenced (Olsen et al., 2016), we searched for proteins that have been implicated in $HCO_{3}^{-}$ uptake in other aquatic photoautotrophs: the anion exchange protein solute carrier type 4 family (SLC4) and carbonic anhydrase in this genome. We performed a BLAST search (https://blast.ncbi.nlm.nih.gov/) with SLC4 isoform 1 from the freshwater plant *Ottelia alismoides* as a query (Huang et al., 2020) and *Z. marina* as the species. For carbonic anhydrase, we searched (https://www.ncbi.nlm.nih.gov/protein/) with carbonic anhydrase as a term and *Z. marina* as the species. Location of all the proteins was determined using WoLF PSORT (https://wolfpsort.hgc.jp/). In contrast, the annotated genome of *P. oceanica* is not currently available and so it was impossible to determine the presence or absence of the genes for these proteins.

### Statistical analysis

Statistical analyses were carried out in Excel for Microsoft 365.

## Results

### Ability to deplete CO_2_ and HCO_3-_ in pH-drift experiments

None of the replicates had reached their final pH after 24 hours; four reached the final pH after 44 hours and four after 63 hours. Both species were able to raise the pH of seawater to over 9.5, depleting the CO_2_ concentration to 0.041 µmol L^-1^ (41 x 10^-6^ mmol L^-1^) in the case of *P. oceanica* and 0.010 µmol L^-1^ (10 x 10^-6^ mmol L^-1^) in the case of *Z. marina* (**Table 1**). This is lower than could be achieved by passive uptake of CO_2_ into the leaf (of the order of 1 µmol L^-1^) and thus indicates an active process, which is likely to be bicarbonate uptake. *Z. marina* had a greater capacity than *P. oceanica* to raise the pH and deplete the concentration of DIC (t-test for difference in final DIC concentration, p<0.05). The final concentration of $\text{HC}{\text{O}}_{3}^{-}$ was also significantly lower in *Z. marina* than in *P. oceanica* (t-test for difference in final $\text{HC}{\text{O}}_{3}^{-}$ concentration, p<0.05). The DIC/Alk quotient, representing the proportion of inorganic carbon remaining at the end of the drift compared to alkalinity, was between 0.5 and 0.44 in the two species, much higher than in many freshwater plants that can use $\text{HC}{\text{O}}_{3}^{-}$ because of the higher concentration of unavailable carbonate ions in seawater compared to fresh water.

**Table 1:** Concentrations of inorganic carbon at the end of pH drift experiments at 20°C in filtered natural seawater.

	pH	Alkalinity	Alkalinity minus borate	[DIC]	[CO_2_]	[ $\text{HC}{\text{O}}_{3}^{-}$ ]	DIC/Alk
		(mequiv L^-1^)		(mmol L^-1^)			
*Posidonia oceanica*	9.56 (0.11)	2.130 (0.113)	1.692 (0.121)	0.872 (0.102)	41 x 10^-6^ (25 x 10^-6^)	0.191 (0.061)	0.51 (0.03)
*Zostera marina*	9.83 (0.06)	1.976 (0.123)	1.521 (0.122)	0.671 (0.066)	10 x 10^-6^ (3 x 10^-6^)	0.088 (0.015)	0.44 (0.02)
Seawater blank	7.89	2.477	2.374	2.189	16 x 10^-3^	1.965	0.96

Molal and molar concentrations were interconverted using a seawater density of 1.027 kg L^-1^. Mean with standard deviation in parentheses, n = 4 apart from the seawater blank that was not replicated. The quotient DIC/Alk is based on the alkalinity minus the borate alkalinity. The mean pH was calculated geometrically.

### Rates of photosynthesis as a function of CO_2_ and HCO_3-_

**Figure 1:**
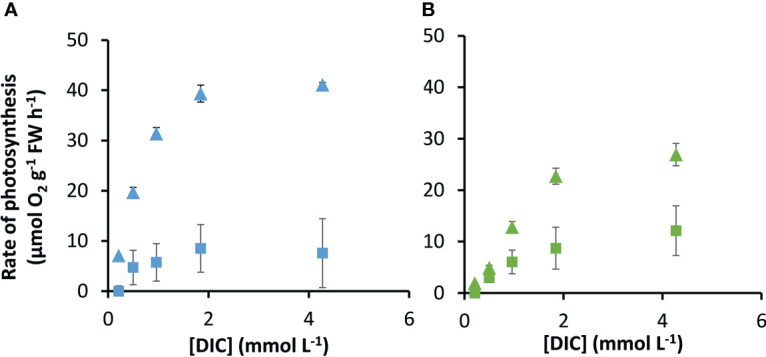
Measured response of photosynthesis by *P. oceanica* and *Z. marina* to concentrations of DIC. Measurements were made at air-equilibrium CO_2_ (squares) and after reducing the pH to 7 (triangles) for *P. oceanica* **(A)** and *Z. marina* **(B)**. Mean and standard deviation of triplicate measurements are shown.

**Figure 2:**
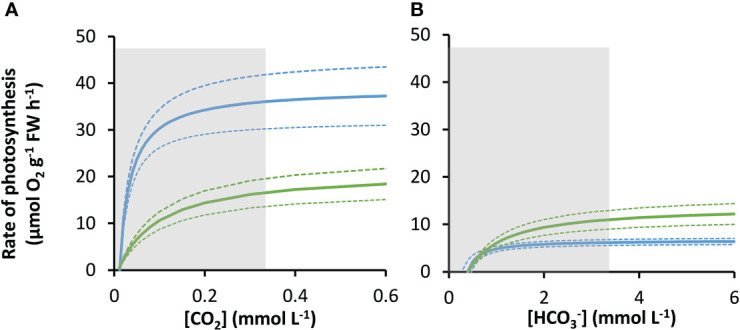
Modelled responses of photosynthesis by *P. oceanica* and *Z. marina* to inorganic carbon. Response of *P. oceanica* (blue) and *Z. marina* (green) to concentration of CO_2_ **(A)** and $\text{HC}{\text{O}}_{3}^{-}$ **(B)**. The bold solid line represents the mean and the upper and the lower dotted lines represent the upper and lower standard deviation. The concentration range of the measurements used to construct the models is indicated by the grey shaded region.

The rates of photosynthesis by *P. oceanica* and *Z. marina* increased with the concentration of DIC and were strongly stimulated when the pH was decreased to pH 7, generating CO_2_ (**Figure 1**), particularly for *P. oceanica*. Rates can be normalized to leaf area, chlorophyll *a* or chlorophyll *a* and *b*, and dry weight using the results in **Supplementary Table 4**. P*. oceanica* had a significantly lower leaf area per g fresh weight and dry weight per g fresh weight than *Z. marina* but a higher chlorophyll content. Results of the modelled kinetic characteristics for CO_2_ and $\text{HC}{\text{O}}_{3}^{-}$ uptake are given in **Table 2** and the modelled responses visualized in **Figure 2**. The total uptake rates at inorganic carbon saturation in the two species were not significantly different on a fresh weight basis, however the maximal CO_2_-dependent rate of photosynthesis in *P. oceanica* was significantly higher than in *Z. marina* and conversely the maximal $\text{HC}{\text{O}}_{3}^{-}$ dependent rate was significantly higher in *Z. marina* than in *P. oceanica* (**Table 2**). Values of K_½_ for both CO_2_ and $\text{HC}{\text{O}}_{3}^{-}$ were significantly lower in *P. oceanica* than in *Z. marina*. The slope of increasing photosynthesis rate at limiting concentration of CO_2_, was significantly higher, by a factor of 7, in *P. oceanica* than in *Z. marina*, but the equivalent slope for $\text{HC}{\text{O}}_{3}^{-}$ was only 1.6-times higher. The slope for $\text{HC}{\text{O}}_{3}^{-}$ uptake was less than 2% that of CO_2_ uptake in *P. oceanica* but 8% in *Z. marina* indicating the greater importance of $\text{HC}{\text{O}}_{3}^{-}$ in the carbon economy of *Z. marina* given that the concentration of $\text{HC}{\text{O}}_{3}^{-}$ is ~140-fold higher than that of CO_2_ in seawater.

**Table 2:** Kinetic characteristics of CO_2_ and HCO_3^-^_ uptake by *P. oceanica* and *Z. marina* determined using the model in equation 1.

Carbon source	Characteristic	*P. oceanica*	*Z. marina*	P:Z
Both	Total $V_{net}^{max}$ (µmol O_2_ g^-1^ FW h^-1^)	45.5 (7.4)	35.1 (6.3)	1.3^NS^
CO_2_	$V_{net}^{max}$ (µmol O_2_ g^-1^ FW h^-1^)	38.8 (6.9)	21.3 (3.8)	1.8^*^
CO_2_	K_½_ (mmol L^-1^)	0.036 (0.007)	0.100 (0.001)	0.4^**^
CO_2_	Compensation point (mmol L^-1^)	0.011 (0.001)	0.010 (0.000)	1.2^**^
CO_2_	Slope (µmol O_2_ g^-1^ FW h^-1^ mmol^-1^ L)	1637 (392)	234 (42)	7.0^*^
$\text{HC}{\text{O}}_{3}^{-}$	$V_{net}^{max}$ (µmol O_2_ g^-1^ FW h^-1^)	6.6 (0.7)	13.9 (2.5)	0.5^*^
$\text{HC}{\text{O}}_{3}^{-}$	K_½_ (mmol L^-1^)	0.615 (0.123)	1.175 (0.001)	0.5^*^
$\text{HC}{\text{O}}_{3}^{-}$	Compensation point (mmol L^-1^)	0.379 (0.104)	0.404 (0.001)	0.9^NS^
$\text{HC}{\text{O}}_{3}^{-}$	Slope (µmol O_2_ g^-1^ FW h^-1^ mmol^-1^ L)	28.2 (3.2)	18.0 (3.3)	1.6^*^
–	R^2^	0.992	0.989	–

Values are the means of three replicates with standard deviation in parentheses. K_½_ is the modelled K_½_ plus the compensation concentration and the slope is calculated from the ratio of $V_{net}^{max}$ to the modelled K_½_ concentration. P:Z is the ratio of values for *P. oceanica* and *Z. marina*. $V_{net}^{max}$ for CO_2_ is: α x Total $V_{net}^{max}$ and $V_{net}^{max}$ for $\text{HC}{\text{O}}_{3}^{-}$ is: (1-α) x Total $V_{net}^{max}$ in Eqn 1. R^2^ is the mean derived from linear regressions between measured and modelled values for each replicate. The significance of the difference between the two species was determined with a t-test and presented as: NS, not significant, *P<0.05, **P<0.01.

**Figure 3:**
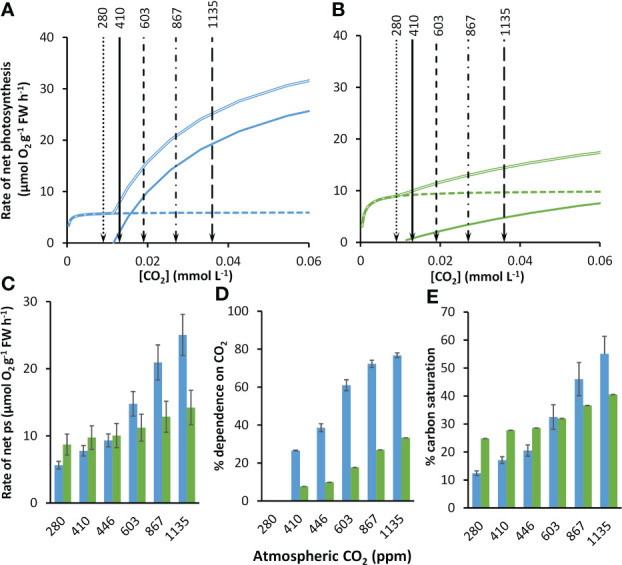
Modelled responses of photosynthesis by *P. oceanica* and *Z. marina* to increasing concentrations of atmospheric CO_2_. Responses of photosynthesis based on CO_2_ (solid line), $\text{HC}{\text{O}}_{3}^{-}$ (dashed line) and the sum of the two (double line) to rising CO_2_ for *P. oceanica* **(A)** and *Z. marina* **(B)**. The atmospheric equivalent (ppm) is given at the top of the panels and indicated by lines and arrows; for clarity the 446 ppm line is not shown. Summary of results for *P. oceanica* (blue) and *Z. marina* (green) showing: rate of net photosynthesis (ps) **(C)**; % dependence on CO_2_ **(D)**; and % inorganic carbon saturation **(E)**. See text for explanation of the atmospheric CO_2_ ppm used. Error bars represent one standard deviation.

As atmospheric CO_2_ partial pressure increases, at equilibrium with the atmosphere, the concentrations of $\text{HC}{\text{O}}_{3}^{-}$ as well as dissolved CO_2_ also increase (**Supplementary Table 3**), markedly stimulating rates of net photosynthesis in both species (**Figures 3A, B** **)**. At equilibrium with 410 ppm, the modelled rate of net photosynthesis in *Z. marina* was about 1.25-times greater than those of *P. oceanica* (**Figure 3C**). The compensation concentration for CO_2_ was slightly below the concentration at equilibrium with 410 ppm in both species (**Table 2**). Nevertheless, CO_2_ was projected to contribute 26.6% to net photosynthesis under these conditions for *P. oceanica*, but only 7.7% for *Z. marina* (**Figure 3D**). The compensation concentration for $\text{HC}{\text{O}}_{3}^{-}$ was about a fifth of the seawater concentration (**Table 2**). $\text{HC}{\text{O}}_{3}^{-}$ was the major source of inorganic carbon under ambient concentrations of CO_2_ and HCO_3_ and was 96% and 65% of the $V_{net}^{max}$ for $\text{HC}{\text{O}}_{3}^{-}$ for *P. oceanica* and *Z. marina* respectively (**Figures 3A, B** **)**. Overall, rates of photosynthesis by *P. oceanica* were 17% carbon-saturated, while those of *Z. marina* were 28% saturated under ambient conditions (**Figure 3E**).

### Modelled responses to past and future concentrations of CO_2_ and HCO_3-_

The kinetic data described above were used to project photosynthesis responses of the two species to the concentrations of CO_2_ and $\text{HC}{\text{O}}_{3}^{-}$ for pre-industrial atmospheric CO_2_ partial pressure (**Supplementary Table 3**). At the pre-industrial CO_2_ partial pressure of 280 ppm, the CO_2_ concentration was below the CO_2_ compensation point and photosynthesis was solely dependent on $\text{HC}{\text{O}}_{3}^{-}$ in both species (**Figure 3D**). Projections were made for a range of future partial pressures for different shared socioeconomic pathway scenarios (**Supplementary Table 3**). From an atmospheric partial pressure of CO_2_ from 410 to 1135 ppm for the SSP5-8.5 scenario the concentration of CO_2_ increased 2.8-fold (by 0.023 mmol L^-1^) and the concentration of $\text{HC}{\text{O}}_{3}^{-}$ increased 1.1-fold (by 0.245 mmol L^-1^). With increasing concentration of CO_2_, rates of CO_2_-dependent photosynthesis increased 9.3-fold and $\text{HC}{\text{O}}_{3}^{-}$ dependent rates increased 1.02-fold in *P. oceanica* so that CO_2_ contributed 77% of net photosynthesis at the highest CO_2_ concentration of 1135 ppm. In contrast, rates of CO_2_-dependent photosynthesis increased 6.3-fold and $\text{HC}{\text{O}}_{3}^{-}$ dependent rates increased 1.1-fold in *Z. marina* and CO_2_ only contributed 33% of net photosynthesis at the highest CO_2_ concentration. Consequently, the increasing CO_2_ concentration benefited net photosynthesis of *P. oceanica* to a much greater extent than *Z. marina* (**Figure 3D**). At 410 ppm the rate of *Z. marina* was 1.25-fold higher than that of *P. oceanica* but despite an increase in rate of photosynthesis in *Z. marina* at the highest CO_2_ scenario of 1135 ppm for SSP5-8.5, the rate of *P. oceanica* was 1.76-fold higher than that of *Z. marina* (**Figure 3D** **)**. As a result of the high dependence of *P. oceanica* on CO_2_, its carbon saturation increased 3.2-fold from 17% at 410 ppm to 55% at 1135 ppm while carbon saturation of *Z. marina* increased 1.5-fold from 28% to 41% over the same range (**Figure 3E**).

### Stable carbon isotopes and discrimination against inorganic carbon sources

**Figure 4:**
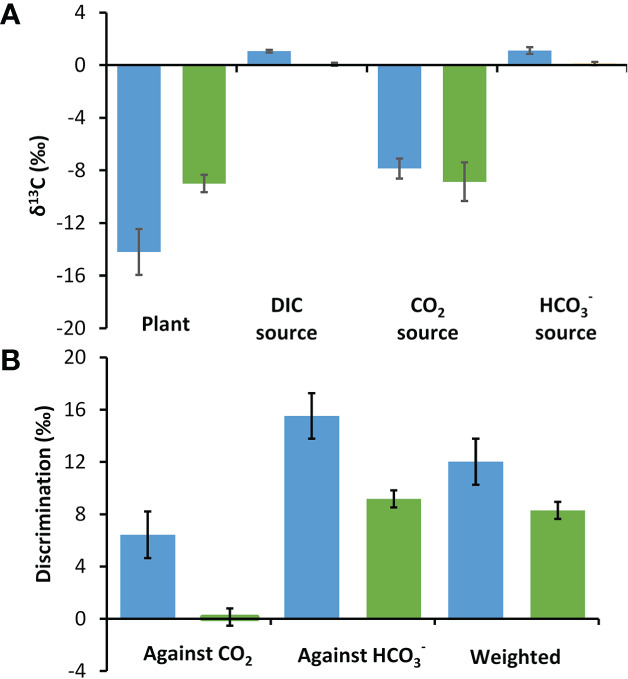
Stable δ^13^C in *P. oceanica* (blue) and *Z. marina* (green) and their potential inorganic carbon sources. **(A)** Measured δ^13^C organic carbon values of the seagrasses and of DIC and calculated CO_2_ and $\text{HC}{\text{O}}_{3}^{-}$ δ^13^C sources at their collection site. **(B)** Discrimination against CO_2_, $\text{HC}{\text{O}}_{3}^{-}$ and the overall discrimination weighted according to estimated proportional use of these two sources of inorganic carbon (**Figure 3C**). Error bars show one standard deviation for five replicates.

Plant δ^13^C values were 5.21‰ more depleted in *P. oceanica* at -14.20‰ than in *Z. marina* at -8.99‰ (t-test, p<0.01) (**Figure 4**). The δ^13^C for DIC at the two collection sites were similar at 1.06‰ at the *P. oceanica* collection site and 0.06‰ at the *Z. marina* collection site. The calculated values of δ^13^CO_2_ at the two sites were -7.86‰ and -8.86‰ and $\delta^{13}\text{HC}{\text{O}}_{3}^{-}$ were 1.11‰ and 0.11‰ respectively. Discrimination was calculated against CO_2_ and $\text{HC}{\text{O}}_{3}^{-}$ individually and also in combination weighted by the estimated contribution of these two carbon sources under ambient conditions using the kinetic model. The discrimination against CO_2_ and $HCO_{3}^{-}$ individually was about 6.3‰ greater in *P. oceanica* than in *Z. marina*. The weighted discrimination of *P. oceanica* at 12.02‰ was 3.72‰ greater than by *Z. marina* at 8.30‰ (**Figure 4**).

### Analysis of the *Zostera marina* genome for putative proteins involved in HCO_3-_ uptake

The *Z. marina* genome contains two sequences of a protein annotated as a boron transporter (KMZ66170 and KMZ71533). When blasted against NCBI Non-redundant protein sequences database, the top 99 proteins retrieved had between 80.3 and 74.7% similarity for KMZ66170 and 70.0 and 65.5% similarity for KMZ71533. The top 99 protein sequences were annotated as a boron transporter (63) or a bicarbonate transporter (6) with the remainder unidentified for KMZ66170 and as a boron transporter (74) or a bicarbonate transporter (2) with the remainder unidentified for KMZ71533. Boron transporters and $\text{HC}{\text{O}}_{3}^{-}$ anion exchange proteins both belong to the SLC4 family (Takano et al., 2002; Thurtle-Schmidt and Stroud, 2016), strongly suggesting that *Z. marina* possesses SLC4. Using WoLF PSORT, the location of both proteins was predicted to be chloroplastic.

The *Z. marina* genome contains ten sequences corresponding to carbonic anhydrase (**Supplementary Table 4**). Of these, two were annotated in NCBI as gamma mitochondrial CA (KMZ56823 and KMZ56166), and a third as chloroplastic (KMZ73424). WoLF PSORT was used to determine the location of all ten sequences: it did not predict the same location for the three sequences where a location is given in NCBI, and both results are presented in **Supplementary Table 5**. Of particular relevance to carbon acquisition, two proteins were predicted to be extracellular (KMZ64507 and KMZ75401).

## Discussion

### Use of HCO_3-_ in *Posidonia oceanic*a and *Zostera marina*

The data presented here confirm that both studied species are able to use $\text{HC}{\text{O}}_{3}^{-}$ since in pH drift experiments, the CO_2_ concentration was driven from 24-times to 100-times below the concentration consistent with diffusive entry of CO_2_ of around 1 µmol L^-1^. The maximum pH generated by *P. oceanica* lies within the range of nine species studied by (Borum et al., 2016) while *Z. marina* has a slightly greater carbon extraction capacity than the species they studied. The final pH for *Phyllospadix scouleri*, 9.76 (Stepien et al., 2016) is intermediate between the two species studied here. The final pH-drift values reported here are greater than those in (Capó-Bauçà et al., 2022). The δ^13^C value for *P. oceanica* reported here at -14.20‰, is less negative than the -16.67‰ reported by (Capó-Bauçà et al., 2022) and more negative than the mean of ten records for this species at -13.5‰ compiled in Appendix S4 of (Stepien, 2015) but within the reported range of -16.20 to -12.1‰. Our value for *Z. marina* of -8.99‰ is less negative than the value of -12.28‰ of Capó-Bauçà et al. (2022) and the mean of 27 values for this species (Appendix S4 of (Stepien, 2015)) but within their measured range of -13.63 to -8.34‰. *Z. marina* appears to be consistently less depleted in ^13^C than *P. oceanica*. This is in agreement with the physiological measurements reported here, suggesting that *Z. marina* has a greater dependence on $\text{HC}{\text{O}}_{3}^{-}$ under ambient conditions than *P. oceanica*. The δ^13^C values for *Z. marina* are too positive for *Z. marina* are too large to be explained by CO_2_ use alone, while those of *P. oceanica* are only just explicable if the use of CO_2_ is very strongly limited by diffusion (Maberly et al., 1992; Raven et al., 2002).

Buffers can substantially increase the K_½_ concentration of $\text{HC}{\text{O}}_{3}^{-}$ uptake in seagrasses (Hellblom et al., 2001): all K_½_ concentrations given here were thus measured in the absence of buffers. At a background CO_2_ concentration of 15 to 19 µmol L^-1^ Invers et al. (2001) reported K_½_ concentrations for $\text{HC}{\text{O}}_{3}^{-}$ of 0.44 mmol L^-1^ for *P. oceanica* and Rubio et al. (2017) found a K_½_ for $\text{HC}{\text{O}}_{3}^{-}$ uptake of 0.35 mmol L^-1^ which is lower than we reported here for this species at 0.62 mmol L^-1^ (**Table 2**). The K_½_ values for $\text{HC}{\text{O}}_{3}^{-}$ for *Z. marina* from the northern Pacific (Invers et al., 2001) at 0.56 mmol L^-1^ and the North Sea at a background CO_2_ concentration of about 8 µmol L^-1^ (Sand-Jensen and Gordon, 1984) at 0.60 mmol L^-1^ are substantially lower than the value of 1.18 mmol L^-1^ reported here (**Table 2**). In contrast, the K_½_ DIC concentration for *Z. marina* from the North Sea off the Swedish coast was 1.60 mmol L^-1^ (Hellblom et al., 2001) is predicted to, under the experimental conditions of pH 8.1, 20°C and a salinity of 30, is equivalent to a $\text{HC}{\text{O}}_{3}^{-}$ concentration of 1.44 mmol L^-1^, slightly greater than our estimate. Recent estimates of K_½_ concentrations of DIC by Capó-Bauçà et al. (2022) at 0.73 mmol L^-1^ for *P. oceanica* and 1.47 mmol L^-1^ for *Z. marina* (roughly 0.63 and 1.26 mmol L^-1^ $\text{HC}{\text{O}}_{3}^{-}$ if the pH was 8.0) are very similar to ours at 0.62 and 1.18 mmol L^-1^ for *P. oceanica* and *Z. marina* respectively. Other seagrass species have also been estimated to have high K_½_ values for $\text{HC}{\text{O}}_{3}^{-}$ such as *Thalassia testudinum* (>1.22 mmol L^-1^; (Durako, 1993)). Despite these differences, the reported K_½_ concentrations for $\text{HC}{\text{O}}_{3}^{-}$ are below ambient concentrations in seawater and even with the higher K_½_ values, such as those reported here, ambient concentrations of $\text{HC}{\text{O}}_{3}^{-}$ are between 65 and 86% saturated for *Z. marina* and *P. oceanica* respectively.

Three possible, not mutually exclusive, mechanisms have been suggested to be involved in $HCO_{3}^{-}$ use in seagrasses (Beer et al., 2002; Beer et al., 2006; Larkum et al., 2017). The first mechanism that is widespread, involves the maintenance of chemical equilibrium concentrations of CO_2_ close to the plasmalemma by a periplasmic carbonic anhydrase that can be inhibited by acetazolamide (Koch et al., 2013; Borum et al., 2016; Capó-Bauçà et al., 2022). Photosynthesis by *P. oceanica* and *Z. marina* are sensitive to the inhibitor acetozolamide (Invers et al., 1999; Hellblom et al., 2001) but *P. oceanica* was inhibited to a greater extent than *Z. marina* (Capó-Bauçà et al., 2022). The presence of two periplasmic CAs (KMZ64507 and KMZ75401) was confirmed in *Z. marina* by our sequence analysis. In this species, a permeable inhibitor, ethoxyzolamide, had an additional inhibitory effect (Capó-Bauçà et al., 2022), consistent with some of the internal carbonic anhydrase sequences we detected in the *Z. marina* genome. The second mechanism involves proton extrusion at the plasmalemma that converts $\text{HC}{\text{O}}_{3}^{-}$ to CO_2_, possibly accelerated by external carbonic anhydrase, and can be inhibited by buffers (Uku et al., 2005; Beer et al., 2006; Borum et al., 2016). *Z. marina* was more strongly inhibited by TRIS buffer than *P. oceanica* (Capó-Bauçà et al., 2022). The third mechanism involves direct uptake of $\text{HC}{\text{O}}_{3}^{-}$ . In *P. oceanica*, proton export by a fusicoccin-sensitive H^+^-ATPase in the plasmalemma is the driving force that allows entry of $\text{HC}{\text{O}}_{3}^{-}$ ions by symport with H^+^ ions (Rubio et al., 2017). It is not clear whether or not a $\text{HC}{\text{O}}_{3}^{-}H^{+}$ symport is operating in *Z. marina*. However, this species has six H^+^-ATPase genes and this enzyme plays a role in salt-tolerance (Muramatsu et al., 2002) but may also be involved in direct or indirect $\text{HC}{\text{O}}_{3}^{-}$ use (Fernandez et al., 1999; Rubio and Fernández, 2019).

In marine macroalgae (Beer, 1994) and the freshwater angiosperm *Ottelia alismoides* (Huang et al., 2020), there is evidence that an anion exchange protein, SLC4, is involved in $\text{HC}{\text{O}}_{3}^{-}$ use since photosynthesis can be inhibited by DIDS (4,4’-diisothiocyanatostilbene-2,2’-disulfonate). In contrast, at pH 8.1 DIDS had no effect on rates of photosynthesis in *Z. marina* (Hellblom et al., 2001; Capó-Bauçà et al., 2022). Our analysis of the *Z. marina* genome shows that it possesses two genes for a SLC4 protein. The discrepancy between the lack of DIDS sensitivity and the presence of SLC4 in the genome could be explained by the predicted location of the two SLC4 proteins in the chloroplast. SLC4 proteins have also been found in the chloroplast membranes of the marine diatom *Phaeodactylum tricornutum* (Matsuda et al., 2017) and predicted in the chloroplast of the freshwater diatom *Asterionella formosa* (Maberly et al., 2021). *Z. marina* differs from *O. alismoides* (Huang et al., 2020) where SLC4 is periplasmic. *P. oceanica* is slightly inhibited by DIDS (Capó-Bauçà et al., 2022) and it will be interesting to determine if SLC4 is present in its genome and where this protein is located.

Freshwater plants that are able to use $\text{HC}{\text{O}}_{3}^{-}$ have a higher K_½_ for CO_2_ and a lower slope of photosynthesis rate *vs* CO_2_ concentration than freshwater plants restricted to CO_2_ (Maberly and Madsen, 1998). This is one of the ‘costs’ of using $\text{HC}{\text{O}}_{3}^{-}$ , in addition to an energy cost (Raven et al., 2014) that gives a disadvantage to using $\text{HC}{\text{O}}_{3}^{-}$ in some ecological situations. The affinities of seagrasses and freshwater plants for CO_2_ based on the slope of photosynthesis *vs* concentration of CO_2_ (**Table 2**) converted to a dry weight basis, using values in **Supplementary Table 4**, were compared. The slope (as µmol g^-1^ DW s^-1^ L mmol^-1^ CO_2_) for *P. oceanica* at 2.51 is similar to the mean for freshwater plants restricted to CO_2_ at 2.41 (Maberly and Madsen, 1998), while the slope for *Z. marina* at 0.33 is four-times lower than the mean slope for freshwater plants able to use $\text{HC}{\text{O}}_{3}^{-}$ at 1.35. For freshwater plants, the lower affinity for CO_2_ in species able to use $\text{HC}{\text{O}}_{3}^{-}$ , compared to those restricted to CO_2_, was caused by a higher internal resistance to CO_2_ exchange (Madsen and Maberly, 2003). This was suggested to reduce losses of CO_2_ from the leaf produced by active uptake of $\text{HC}{\text{O}}_{3}^{-}$ .

### Seagrasses in past and future climates

Ocean warming and the increased frequency and intensity of extreme events, such as heatwaves and storms, are likely to be detrimental to seagrass meadows (Marba and Duarte, 2010; Hammer et al., 2018; Oprandi et al., 2020) along with interactions with other processes such as anthropogenic nutrient stress (Helber et al., 2021). In contrast, increasing CO_2_ concentrations at the surface of the ocean may help to increase seagrass productivity (Hall-Spencer et al., 2008; Jiang et al., 2010; Koch et al., 2013; Burnell et al., 2014; Garrard and Beaumont, 2014; Ow et al., 2015; Pajusalu et al., 2016). Borum et al. (Borum et al., 2016) showed that rates of net photosynthesis increased with CO_2_ concentration from 9 to 24 µmol L^-1^ in seven of the nine species studied, but none significantly. However, there was a statistically significant increase in all except one species when CO_2_ was increased to 275 µmol L^-1^. In our model, the photosynthesis rate of *Z. marina* is predicted to increase 1.46-fold from 9 to 36 µM CO_2_ as a result of a 9.29-fold and 1.02-fold increase in the CO_2_ and $\text{HC}{\text{O}}_{3}^{-}$ -dependent rate respectively. The overall increase is similar to the increase of three species of tropical seagrasses of 1.49-fold to 1.68-fold over a similar CO_2_ concentration range based on the regressions of photosynthesis rate to CO_2_ partial pressure in Ow et al. (2015). In contrast, the photosynthesis rate of *P. oceanica* would increase 3.22-fold from 9 to 36 µM CO_2_ as a result of a 6.32-fold and 1.05-fold increase in the CO_2_ and $\text{HC}{\text{O}}_{3}^{-}$ -dependent rate respectively. This suggests that this species may benefit particularly from rising concentrations of CO_2_ (**Figure 3**). This is the result of a higher affinity and capacity to use CO_2_, but a lower capacity to use $\text{HC}{\text{O}}_{3}^{-}$ in *P. oceanica* than in *Z. marina* (**Table 2**). Unlike *Z. marina*, future projections suggest that CO_2_ will become the major source of inorganic carbon in *P. oceanica* (**Figure 3**).

Although increased atmospheric CO_2_ concentration will increase the concentration of CO_2_ in water, increased water temperature will reduce CO_2_ solubility by about 5% for a temperature increase of 2°C. Greater effects reducing the benefit of increased inorganic carbon availability are likely to result from interaction with other environmental factors. For example, limitation of photosynthesis by light or temperature (Maberly, 1985) or nutrients will decrease inorganic carbon limitation. In addition, Rubio et al. (2020) showed that there was an interaction between nitrate uptake and bicarbonate uptake in *P. oceanica*. High concentrations of $\text{HC}{\text{O}}_{3}^{-}$ led to loss of cytosolic nitrate *via* S-type anion channels, which could lead to, or increase, nitrogen limitation.

A mystery surrounds the reason for the very small numbers of plant species in the ocean. Suggestions include difficulties of pollination, especially given the lack of co-evolution with insects (Van der Hage, 1996), competition with macroalgae (Maberly and Gontero, 2018), toxicity of sulfide in the sediment (van der Heide et al., 1996) but not salinity *per se* (Touchette, 2007). Seagrasses are believed to have invaded the ocean about 100 to 140 Mya, probably from freshwater descendants given their phylogeny (Les et al., 1997; Les and Tippery, 2013). At this time, the atmospheric CO_2_ partial pressure is believed to have been 860 to 1000 ppm (Foster et al., 2017) while ocean carbonate alkalinity was similar to that today (Zeebe and Tyrrell, 2019). Assuming an unchanged physiology, CO_2_ concentrations in the Cretaceous will have been slightly more favourable for seagrasses than those today. Ironically, these concentrations are similar to some projections for the end of the century. While CO_2_ concentrations in the oceans are close to atmospheric equilibrium, in freshwaters, especially rivers, CO_2_ concentrations can be extremely depleted, but more frequently exceed atmospheric CO_2_ equilibrium (Cole et al., 1994; Maberly et al., 2013). Air-equilibrium concentrations of CO_2_ in the ocean may therefore have reduced the fitness of plants invading from fresh waters.

### Perspectives

A number of physiological uncertainties need to be addressed. First, further work is needed to determine if the different reported K_½_ concentrations for $\text{HC}{\text{O}}_{3}^{-}$ (in the absence of buffers) are the result of genotypic or phenotypic differences in the plant material, the measurement method or how the data were analysed. Secondly, further research is needed to determine the extent to which seagrasses can acclimate to variable CO_2_. Rapid acclimation would be one explanation for the lower $\text{HC}{\text{O}}_{3}^{-}$ compensation during the pH-drift of up to 60 hours compared to the concentrations determined from the short-term kinetic measurements. Thirdly, although there is strong circumstantial evidence for a CCM in many seagrass species, evidence is currently lacking that shows that the concentration of CO_2_ around Rubisco is elevated above the external concentration (Larkum et al., 2017). Fourthly, further work is needed to establish if an anion exchange protein such as SLC4 is involved in the photosynthesis of *Z. marina* and other species of seagrass and where it is located. Fifthly, it is unclear why the affinity to CO_2_ of seagrasses that are able to use $\text{HC}{\text{O}}_{3}^{-}$ , differs among themselves and from freshwater plants. Finally, more research is needed to compare structural difference between the leaves of seagrasses and freshwater plants and the mechanisms and location where $\text{HC}{\text{O}}_{3}^{-}$ is converted to CO_2_ and how this interacts with the different carbonate chemistry in the oceans compared to fresh waters.

The differential ability of seagrasses to exploit inorganic carbon reserves may control their future ecological success. The growing availability of genomic data for seagrasses (Olsen et al., 2016) is becoming a powerful tool to understand differential species responses to environmental change, especially when combined with more traditional approaches (Davey et al., 2016). This will provide more insights into the mechanisms supporting inorganic carbon uptake of individual species and increase our understanding of the different carbon uptake capabilities among species and their ecological consequences under environmental change.

## Data availability statement

The original contributions presented in the study are included in the article/**Supplementary Material**. Further inquiries can be directed to the corresponding author.

## Author contributions

SM and BG devised the work plan and carried out the experiments. AS made the stable carbon isotope measurements. SM and BG analyzed the data and wrote the manuscript. All authors contributed to the article and approved the submitted version.

## Funding

BG is supported by the Centre National de la Recherche Scientifique, Aix-Marseille Université, and currently by the Agence Nationale de la Recherche (OCEANIA, ANR-21-CE20-0029-01). SM’s work was supported by a visiting scholarship from the University of Aix-Marseille and a ‘Make our Planet Great Again’ scholarship from the French Government.

## Acknowledgments

We thank Sandrine Ruitton for help and advice and for collecting *P. oceanica* with the crew of the Antedon II and the CNRS divers. Veronique Receveur-Brechot is thanked for help with field work.

## Conflict of interest

The authors declare that the research was conducted in the absence of any commercial or financial relationships that could be construed as a potential conflict of interest.

## Publisher’s note

All claims expressed in this article are solely those of the authors and do not necessarily represent those of their affiliated organizations, or those of the publisher, the editors and the reviewers. Any product that may be evaluated in this article, or claim that may be made by its manufacturer, is not guaranteed or endorsed by the publisher.
