# Supplementary material for: The differential ability of two species of seagrass to use carbon dioxide and bicarbonate and their modelled response to rising concentrations of inorganic carbon
Source: Front Plant Sci. 2022 Sep 29;13:936716. doi: 10.3389/fpls.2022.936716 (PMC9648567; doi:10.3389/fpls.2022.936716)
Supplement: Supplementary file 1 [file DataSheet_1.pdf]

SUPPLEMENTARY TABLE 1 | Composition of the artificial seawater.

Salt	Concentration (mmol L^{-1})
NaCl	419.6
Na_2SO_4	28.9
$MgCl_2.6H_2O$	25.6
KCl	9.3
$CaCl_2.2H_2O$	8.0
KBr	2.4

SUPPLEMENTARY TABLE 2 | Calculated concentrations of DIC and inorganic carbon species for different carbonate alkalinities at equilibrium with 410 ppm CO_2 and after acidifying to pH 7 used in the kinetic experiments. The volume of HCl added to 2 mL of artificial seawater was determined from a linear regression between volume of acid and alkalinity (volume of acid (µL) = 3.512 (0.066) alkalinity (mequiv L^{-1}) + 0.329 (0.162); R^2 = 0.999) where values represent one standard error.

			mmol L^{-1}			
Initial Alkalinity (mequiv L^{-1})	Vol 0.1 M HCl added to chamber (µl)	pH	[DIC]	[CO_2]	[HCO_3^-]	[CO_3^{2-}]
		After equilibrium with air				
0.2	0	7.07	0.210	0.013	0.195	0.002
0.5	0	7.46	0.499	0.013	0.473	0.013
1	0	7.74	0.963	0.013	0.902	0.048
2	0	8.01	1.845	0.013	1.669	0.164
5	0	8.33	4.273	0.013	3.529	0.731
		After acidification				
0.2	1.5	7.00	0.210	0.015	0.193	0.002
0.5	3.0	7.00	0.499	0.036	0.458	0.004
1	5.5	7.00	0.963	0.069	0.885	0.009
2	10.5	7.00	1.845	0.132	1.697	0.016
5	2.6*	7.00	4.273	0.305	3.930	0.038

* 1 M HCl

SUPPLEMENTARY TABLE 3 | pH and concentrations of CO_2 and HCO_3^-, and their ratio, calculated using the CO2SYS_calc program (Pierrot and Wallace, 2006) for past, present and future (2100) atmospheric CO_2 partial pressures relating to different shared socioeconomic pathways (SSPs) based on (Meinshausen et al., 2020). Values are calculated for 20°C, an alkalinity of 2.228 mequiv L^{-1} and a salinity of 36.

Scenario	Atmosphere CO_2 (ppm)	pH	CO_2 (mmol L^{-1})	HCO_3^- (mmol L^{-1})	Ratio HCO_3^-: CO_2
Past	280	8.17	0.009	1.705	189
Present	410	8.03	0.013	1.830	141
SSP1-2.6	446	8.00	0.014	1.855	133
SSP2-4.5	603	7.89	0.019	1.939	102
SSP3-7.0	867	7.75	0.027	2.023	75
SSP5-8.5	1135	7.64	0.036	2.075	58

SUPPLEMENTARY TABLE 4 | Morphological and pigment characteristics of *P. oceanica* and *Z. marina*. Values are the means of three replicates (17 replicates for fresh weight to dry weight) with standard deviation in parentheses. The ratio of *P. oceanica* to *Z. marina* (P:Z) is given and the significance of the difference between the two species was determined with a t-test and presented as: NS, not significant, * P<0.05, ** P<0.01.

Characteristic	*P. oceanica*	*Z. marina*	P:Z
2-sided area per g FW ($cm^2\ g^{-1}$)	141.3 (11.0)	181.0 (10.4)	0.78 (0.1)*
Dry weight per fresh weight ($g\ g^{-1}$)	0.181 (0.006)	0.197 (0.017)	0.92 (0.1)**
Chl*a* per g FW ($\mu g\ g^{-1}$)	711 (71)	405 (44)	1.76 (0.3)**
Chl*a* :Chl*b*	1.72 (0.06)	1.76 (0.12)	0.98 (0.1)^{NS}

SUPPLEMENTARY TABLE 5 | Protein ID and predicted location of carbonic anhydrase proteins in *Z. marina* determined using WoLF PSORT. Locations are: chloroplast (Chlo); extracellular (Extr); nucleus (Nucl); cytoplasm (Cyto); mitochondria (Mito); endoplasmic reticulum (E.R.); golgi (Golgi); vacuole (Vacu); plasma membrane (Plas). Proteins that shuttle between two locations are given dual localisation separated by an 'underscore'. The number of proteins of known localization with the most similar localization features to the query is given; the highest number(s) for each sequence are highlighted in bold and underlined. Sequences in red were also given a location in NCBI (https://www.ncbi.nlm.nih.gov/protein/), which is indicated by grey shading and underlined. The total number of carbonic anhydrase proteins in each location based on the most similar localization is given, with values in parentheses taking into account the different annotated locations.

	Predicted Location												
Protein ID	Chlo	Extr	Nucl	Cyto	Mito	E.R.	Golgi	Vacu	Plas	E.R._plas	Mito_plas	Golgi_plas	Cyto_Nucl
KMZ59859		1	1	**4**	1	3.5			3.5	**4**			
KMZ63672		3		2		2		**7**					
KMZ64507		**7**	3		1.5	1		1			1.5		
KMZ69362	2	1	1	**3**	2	1		2	2				
KMZ71968	2	2	1	1	1	1	1	**5**					
KMZ73035	**8**	3	2	1									
KMZ75401	1	**5**	1	1		1	1	4					
KMZ56823	**7**	1		2	2			1	1				
KMZ56166	**6**		2	2	1			2	1				
KMZ73424	4		**6.5**		2							1	4
**Number**	**3 (4)**	**2**	**1**	**2 (4)**	**0 (2)**	**0**	**0**	**2**	**0**	**1**	**0**	**0**	**0**

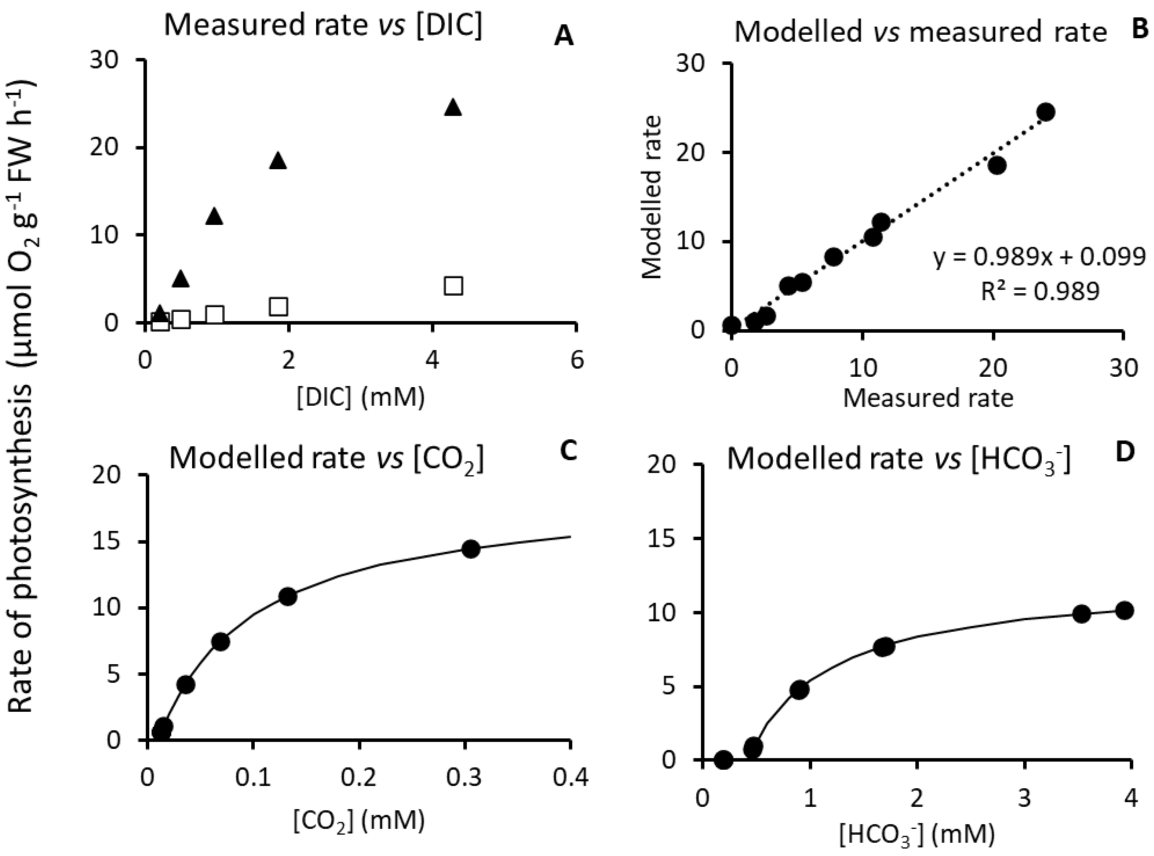

SUPPLEMENTARY FIGURE 1 | Example results for one replicate of *Z. marina***.** A, Measured rate of net photosynthesis versus the concentration of inorganic carbon (DIC) at air-equilibrium (open squares) and after acidification to pH 7.0 (closed triangle). B, Modelled versus measured rate of net photosynthesis with linear regression of the relationship. C, Modelled rate of net photosynthesis versus concentration of CO_2. D, Modelled rate of net photosynthesis versus concentration of HCO_3^-.

REFERENCES

Meinshausen, M., Nicholls, Z.R.J., Lewis, J., Gidden, M.J., Vogel, E., Freund, M. et al. (2020). The shared socio-economic pathway (SSP) greenhouse gas concentrations and their extensions to 2500. *Geosci. Model Dev.* 13**,** 3571-3605.

Pierrot, D., Lewis, E., and Wallace, D.W.R. (2006). MS Excel Program Developed for CO_2 System Calculations. ORNL/CDIAC-105a. Carbon Dioxide Information Analysis Center, Oak Ridge National Laboratory, U.S. Department of Energy, Oak Ridge, Tennessee. doi: 10.3334/CDIAC/otg.CO2SYS_XLS_CDIAC105a
